# Hydroxamic Acid as a Potent Metal-Binding Group for Inhibiting Tyrosinase

**DOI:** 10.3390/antiox11020280

**Published:** 2022-01-29

**Authors:** Joonhyeok Choi, Trilok Neupane, Rishiram Baral, Jun-Goo Jee

**Affiliations:** Research Institute of Pharmaceutical Sciences, College of Pharmacy, Kyungpook National University, 80 Daehak-ro, Buk-gu, Daegu 41566, Korea; crowz124@naver.com (J.C.); ozitrilok99@gmail.com (T.N.); rishirambaral1996@gmail.com (R.B.)

**Keywords:** cheminformatics, docking simulation, histone deacetylase, hydroxamic acid, tyrosinase

## Abstract

Tyrosinase, a metalloenzyme containing a dicopper cofactor, plays a central role in synthesizing melanin from tyrosine. Many studies have aimed to identify small-molecule inhibitors of tyrosinase for pharmaceutical, cosmetic, and agricultural purposes. In this study, we report that hydroxamic acid is a potent metal-binding group for interacting with dicopper atoms, thereby inhibiting tyrosinase. Hydroxamate-containing molecules, including anticancer drugs targeting histone deacetylase, vorinostat and panobinostat, significantly inhibited mushroom tyrosinase, with inhibitory constants in the submicromolar range. Of the tested molecules, benzohydroxamic acid was the most potent. Its inhibitory constant of 7 nM indicates that benzohydroxamic acid is one of the most potent tyrosinase inhibitors. Results from differential scanning fluorimetry revealed that direct binding mediates inhibition. The enzyme kinetics were studied to assess the inhibitory mechanism of the hydroxamate-containing molecules. Experiments with B16F10 cell lysates confirmed that the new inhibitors are inhibitory against mammalian tyrosinase. Docking simulation data revealed intermolecular contacts between hydroxamate-containing molecules and tyrosinase.

## 1. Introduction

Melanin, a high-molecular-weight pigment produced mainly in melanocytes, plays a fundamental role in living beings [1,2,3]. Melanin can protect the skin from ultraviolet light by dissipating >99.9% of the light. Darkness in skin and hair color depends on the amount of melanin. Tyrosinase is a central enzyme in melanin production. Tyrosinase catalyzes the conversion of tyrosine, the substrate for melanin biosynthesis, into dihydroxyphenylalanine (DOPA) and subsequently into dopaquinone, which then spontaneously changes into melanin. Tyrosinase, catechol oxidase, and hemocyanin comprise type-3 copper proteins. Of the two conversions by tyrosinase, monophenol to diphenol and diphenol to quinone, catechol oxidase can only catalyze the second reaction. In contrast, the primary role of hemocyanin is to carry oxygen in some invertebrates. The proteins possess six histidine residues and two juxtaposed copper ions as metal cofactors that are evolutionarily conserved. The three histidine residues form coordinate bonds with the copper ion.

Efforts are ongoing to discover small molecules that modulate the function of tyrosinase for pharmaceutical, cosmetic, and agricultural purposes [4,5,6,7,8,9,10,11]. Tyrosinase inhibitors can be broadly categorized into two groups based on the metal-binding group (MBG) of the compounds: polyphenol and thione-containing molecules. Natural products, such as kojic acid, arbutin, and tropolone, belong to polyphenol inhibitors, whereas phenylthiourea (PTU) and its synthetic analogs comprise thione-containing inhibitors [12]. A structure-based docking screen discovered tetrazole- and triazole-containing compounds as tyrosinase inhibitors with new MBGs [13].

Although it is present in trace amounts, metal is essential for living beings. More than one-quarter of all proteins are metalloproteins, and approximately one-half of all enzymes need metals for catalysis [14,15]. Nevertheless, the development of small molecules that inhibit metalloenzymes through direct binding has lagged behind. Only seven classes of metalloenzymes have been targeted by approximately 70 FDA-approved drugs. The targets (metals) include carbonic anhydrase (Zn^2+^), histone deacetylase (HDAC; Zn^2+^), angiotensin-converting enzyme (Zn^2+^), HIV-1 integrase (Mg^2+^), matrix metalloproteinase (Zn^2+^), lipoxygenase (Fe^2+^), and lanosterol 14α-demethylase (Fe^2+^) [16]. The main concern in developing inhibitors for metalloenzymes is enzyme selectivity. Small-molecule metalloenzyme inhibitors consist of an MBG and other parts for selective interaction with the protein. Due to insufficient selectivity, certain inhibitors can bind to several metalloenzymes, causing unexpected physiological effects through unwanted polypharmacological interactions. Therefore, controlling off-target effects is crucial. However, whether other metalloenzyme inhibitors inhibit tyrosinase is less studied, at least partially due to the relative difficulty in interpreting dicopper atoms in a theoretical and general way.

Hydroxamic acid is one of the most extensively studied MBGs. After Losson’s publication in the 1860s, studies on chemicals containing hydroxamic acid have accumulated. Hydroxamic acid can chelate several metals, which is a feature used for developing hydroxamate-containing metalloenzyme inhibitors. In contrast, natural products, such as siderophores, possess hydroxamic acid as a functional group through which host microorganisms obtain iron from the environments [17]. Some hydroxamic-acid derivatives are also known as antioxidants [18,19,20].

We have discovered tyrosinase inhibitors using biochemical, biophysical, and computational methods [13,21,22,23,24]. In this study, we report that hydroxamic acid can be a general and strong MBG for inhibiting tyrosinase. Combined approaches comprising biochemical, biophysical, and cell-based assays showed that several hydroxamate-containing molecules, including vorinostat, can inhibit tyrosinase. Computational studies using docking simulation and cheminformatics were used to assess detailed features.

## 2. Materials and Methods

Enzyme-activity assays using inhibitors—Cayman Chemical (Ann Arbor, MI, USA), Sigma-Aldrich (St. Louis, MO, USA), and Tokyo Chemical Industry (Tokyo, Japan) provided all the reagents used in this study. The reaction solution for the enzyme assay contained 5 nM mushroom tyrosinase from *Agaricus bisporus* and inhibitors in phosphate-buffered saline containing 5% dimethyl sulfoxide and 0.01% (*w*/*v*) Triton X-100. After 10 min of incubation at 30 °C, 500 μM L-DOPA was added into the mixture as the substrate. The absorbance change induced by the chromogenic product, dopachrome, was measured at 475 nm using Epoch2 from BioTek (Winooski, VT, USA). After confirming the inhibition at a single concentration of 50 μM, the IC_50_ value was calculated by using the inhibitor at a series of different concentrations. The determined IC_50_ value was converted into the inhibitory constant, K_i_, using the Cheng–Prusoff equation with K_m_ [25]. All the fittings and statistical analyses were conducted using MATLAB from MathWorks (Natick, MA, USA).

Differential scanning fluorimetry—Differential scanning fluorimetry (DSF) was used to characterize the direct binding of inhibitors to tyrosinase. In the absence and presence of 500 μM inhibitor, SYPRO^®^ Orange was added to the enzyme solution. The temperature was increased from 30 °C to 85 °C, and the dye fluorescence was measured at excitation and emission wavelengths of 492 and 610 nm, respectively. Time- and temperature-dependent signals were observed using the RT-PCR CFX96 system from BioRad (Hercules, CA, USA). The following equation was nonlinearly minimized to determine the mid-point melting temperature (T_m_).

$$I\left(T\right)=LL+\frac{UL-LL}{\left[1+\exp(\frac{Tm-T}{a})\right]}$$

where *UL* and *LL* indicate the top and baseline of the curves, respectively, and *a* indicates the steepness of the slope. The signals in the range of 33 °C to 66 °C were used for the fitting.

Enzyme-inhibitory kinetics—Upon varying the substrate and inhibitor concentrations, the rates of product generation were measured. Simultaneous nonlinear fitting of all the profiles in an inhibitor was performed by minimizing the ꭓ^2^-value, the sum of the squared differences in theoretical and experimental values, in the Michaelis–Menten equations assuming competitive, uncompetitive, noncompetitive, and mixed models. The fits resulted in the kinetic parameters V_max_, K_m_, K_ic_, and K_iu_ in each model. Reduced ꭓ^2^-values, ꭓ^2^-values divided by the difference in the numbers of input data and fitting parameters, were compared between the models based on the F-test to identify the most appropriate model [26].

Cell-lysate-based activity assays with inhibitors—Cell-lysate-based assays were performed using B16F10 murine melanoma cells obtained from the Korean Cell Line Bank (Seoul, Korea). The B16F10 cells were grown to a density of 1 × 10^5^ cells in Dulbecco’s modified Eagle’s medium supplemented with 10% fetal bovine serum and 1% penicillin−streptomycin at 37 °C under 5% CO_2_. The cells were incubated for 48 h after the addition of 100 μM 3-isobutyl-1-methylxanthine. The cell extracts having tyrosinase activity were prepared.

Cheminformatics and modeling of 3D complex structures—The ChEMBL database [27,28] was used to extract data on the target-specific small bioactive molecules. The protein and ligand complexes were extracted using the PDBbind [29] or the RCSB [30] databases. Intermolecular interactions in the complex structure or the docked models were analyzed and visualized using the OpenEye Grapheme package. Three-dimensional models of complexes formed between mushroom tyrosinase and inhibitors were generated using DOCK 3.7 [31,32], Fred [33,34], and Glide-SP [35,36] with the default settings. The molecules for docking were prepared using LigPrep equipped with Epik from the Schrödinger software.

## 3. Results

### 3.1. Vorinostat and Hydroxamate-Containing HDAC Inhibitors Are Inhibitory for Tyrosinase

The ChEMBL database [27,28] includes 576 small-molecule mushroom tyrosinase inhibitors smaller than 500 Da and more potent than 50 μM in the K_i_ or IC_50_. The molecules include two hydroxamate-containing molecules, CHEMBL2332235 and CHEMBL1825209 (Figure S1). However, the two molecules possess phenolic moieties that mimic the intrinsic substrates, tyrosinase and L-DOPA. Therefore, the phenolic moiety can act as an MBG to directly interact with tyrosinase. An extensive survey indicated three reports on tyrosinase inhibition by hydroxamate-containing molecules [37,38,39]. The compounds include glycine hydroxamate [37], HCA-Phe-NHOH and HCA-Pro-NHOH [38], and caffeoyl-amino acidyl-hydroxamic acid [39]. Except for glycine hydroxamate, the others also contain phenolic moieties (Figure S1).

**Figure 1:**
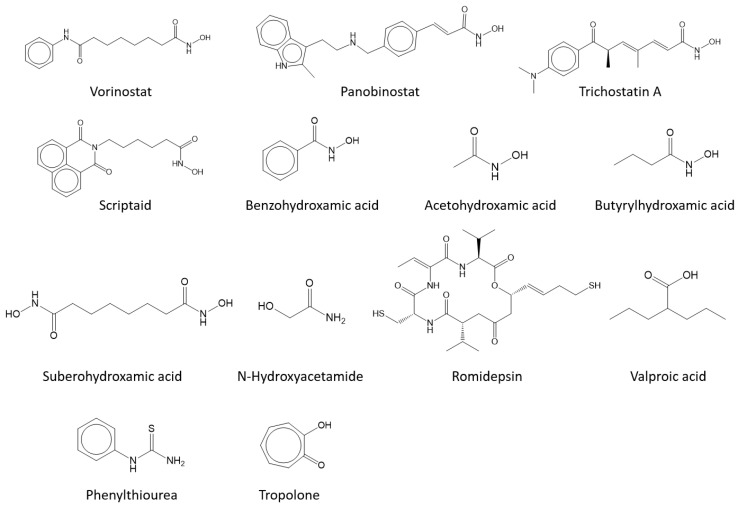
Tested molecules. Of the 13 molecules, phenylthiourea and tropolone are positive controls.

Glycine hydroxamate inhibited tyrosinase with comparable inhibitory activity to arbutin [37]. However, a study has reported that vorinostat (suberanilohydroxamic acid), an FDA-approved anticancer drug that inhibits HDAC, showed little inhibition against mushroom tyrosinase [40]. This disagreement motivated us to evaluate whether vorinostat and other hydroxamate-containing molecules inhibit tyrosinase (Figure 1 and Table 1).

**Figure 2:**
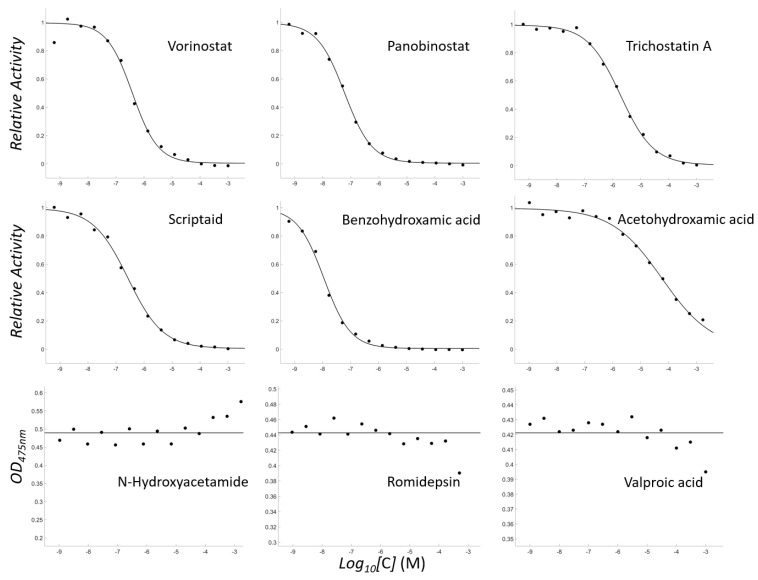
Concentration-dependent inhibitory profiles of the tested molecules. Of the tested molecules, the profiles of 9 molecules are shown. The activities are scaled to have a relative value of 0–1 for vorinostat, panobinostat, trichostatin A, scriptaid, benzohydroxamic acid, and acetohydroxamic acid. The optical densities at 457 nm at the final time interval are presented for N-hydroxyacetamide, romidepsin, and valproic acid.

Notably, vorinostat significantly inhibited mushroom tyrosinase with a K_i_ of 257 nM (Figure 2). We performed the enzyme assays with a series of hydroxamate-containing molecules. The results revealed that an FDA-approved HDAC inhibitor, panobinostat, is a more potent tyrosinase inhibitor than vorinostat, with K_i_ = 40 nM. Other hydroxamate-containing HDAC inhibitors such as trichostatin A, scriptaid, suberohydroxamic acid, and benzohydroxamic acid were all inhibitory, with K_i_ values ranging from 7 nM to 1 μM. Of these, benzohydroxamic acid was the strongest. The inhibition was even more potent than that of the known strong inhibitors PTU and tropolone (Table 1).

Then, we tested with simpler molecules, acetohydroxamic acid and butyrylhydroxamic acid, to confirm that the existence of hydroxamic acid is a sufficient condition for the inhibition. Both inhibited tyrosinase, with K_i_ values of 38 and 32 μM, respectively; however, the degree of inhibition was weaker than that for other drug-like molecules. We also tested N-hydroxyacetamide, which has an identical atomic composition to acetohydroxamic acid but a different topology. It scarcely inhibited tyrosinase.

We next investigated whether HDAC inhibitors with MBGs other than hydroxamic acid could inhibit tyrosinase. We used romidepsin [42] and valproic acid [43], which have sulfide and carboxylate groups as MBGs, respectively. Both inhibited very weakly compared with acetohydroxamic acid (Figure 2), although sulfide and carboxylate are two MBGs present in tyrosinase inhibitors.

Our biochemical data demonstrate that hydroxamic acid is indispensable for HDAC inhibitors to inhibit tyrosinase; however, other parts and their geometric location are equally crucial for potentiating their activities. Here, we would like to note two things. First, the reaction mixture for the enzyme-based assay included 0.01% Triton X-100 to minimize the detection of false positives due to colloidal aggregation [44,45,46,47]. Second, the enzyme concentration was adjusted to be sufficiently low, enabling the quantification of a potent inhibitor.

### 3.2. Biophysical Experiments Confirmed the Direct Interaction between Hydroxamate-Containing Molecules and Tyrosinase

We used differential scanning fluorimetry (DSF) to verify whether active small molecules identified by biochemical assays bound to tyrosinase directly. DSF was used to evaluate the fluorescence profiles of the dye SYPRO^®^ Orange in a mixture with the protein upon elevating the temperature. The alteration in the unfolding process in the presence of a direct binder can shift the DSF pattern. Our findings revealed that mushroom tyrosinase inhibitors could change DSF patterns, where inhibitor addition lowered the T_m_ of the tyrosinase [22].

**Figure 3:**
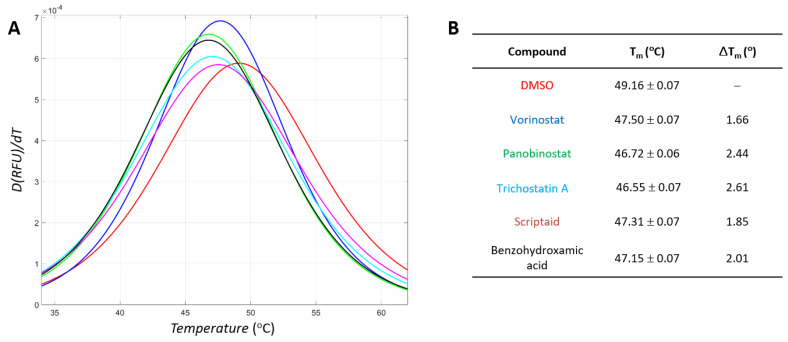
Profiles of differential scanning fluorimetry with tyrosinase and hydroxamate-containing inhibitors. Identical colors are used for the profiles and information for each compound. (**A**) Profiles. Derivatives of the relative fluorescence unit (RFU) by temperature (*D(RFU)/dT*) were constructed in the range of 34 °C to 62 °C. (**B**) Calculated values. Means and standard deviations were obtained from three repeated independent experiments.

We observed pattern changes in the presence of hydroxamate-containing tyrosinase inhibitors. The addition of inhibitors shifted the T_m_ toward a lower temperature. The DSF profiles are shown in Figure 3. The ordered ΔT_m_ are 2.61 degrees for trichostatin A, 2.44 for panobinostat, 2.01 for benzohydroxamic acid, 1.85 for scriptaid, and 1.66 for vorinostat (Figure 3). The ΔT_m_ values were >3 × standard deviation of those without an inhibitor, indicating statistical significance. Moreover, the change was concentration-dependent for each inhibitor. Our data show that direct binding leads to inhibition.

### 3.3. Enzyme Inhibitory Kinetic Experiments Characterize the Modes of the Inhibition

**Figure 4:**
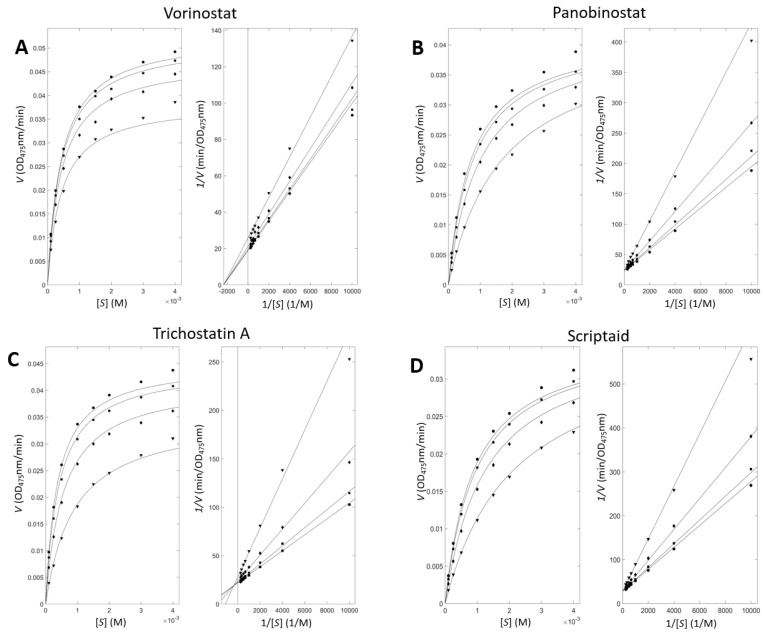

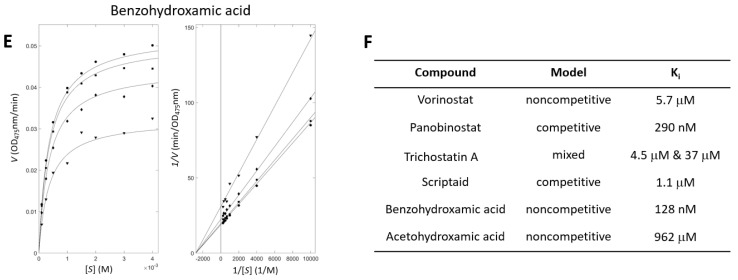
Enzyme-inhibitory kinetics with tyrosinase and inhibitors. (**A**–**E**) Left and right panels show Michaelis–Menten and Lineweaver–Burk plots, respectively, for the five representative inhibitors: ●, ■, ♦ and ▼ represent inhibitor concentrations of 100, 250, 750, and 2250 nM for vorinotstat; 20, 50, 150, and 450 nM for panobinostat; 0.5, 1.2, 3.6, and 10.8 μM for trichostatin A; 100, 200, 600, and 1800 nM for scriptaid; and 5, 10, 30, and 90 nM for benzohydroxamic acid, respectively. (**F**) Kinetics models and simulated inhibitory constants for each inhibitor. The first and second values for trichostatin A mean the inhibitory constants for apo and holo states, respectively.

The inhibitory kinetic experiments provide information for understanding the inhibition by small molecules. The rates of product generation were measured for a series of paired substrate and inhibitor concentrations. Subsequent nonlinear fitting using the Michaelis−Menten equations extended with inhibitory models and constants produced the inhibition mode and the binding constant. Figure 4 shows the kinetic profiles with hydroxamate-containing tyrosinase inhibitors. The apparent closeness in the fitted and experimental points in both the Michaelis−Menten and the Lineweaver−Burk plots validates our analyses.

Figure 4F lists the parameters for inhibition. The order of the simulated values is qualitatively consistent with the order of the experimental K_i_ values. However, one should be careful not to overinterpret the simulated modes and K_i_ values. The modes were chosen using the F-test, comparing reduced χ^2^-values from each model. The differences are often unclear from the available data points.

### 3.4. Hydroxamate-Containing Molecules Were Inhibitory for Mammalian Tyrosinase as Well

Next, we tested whether hydroxamate-containing tyrosinase inhibitors inhibited mammalian tyrosinase. The comparison of mushroom tyrosinase (UniProt ID: C7FF04 and PDB ID: 2Y9X), and AlphaFold-generated human (P14679) and mouse (P11344) tyrosinase structures [48,49] reveals that the sequence identities in the structure-based alignment to mushroom tyrosinase are 17.4 and 19.0% for human and mouse tyrosinases. It suggests that their similarities are limited. Indeed, the aligned structures of mushroom and mammalian tyrosinases illustrate the large differences (Figure S2). The root-mean-square-deviation value (RMSD) in the structural region between mushroom and human (mouse) tyrosinases is 4.14 (4.20) Å, with a TM-score [50] of 0.608 (0.608). Nevertheless, the regions coordinating two copper atoms are highly conserved, reflecting evolutionary pressure. Meanwhile, the RMSD between the AlphaFold-generated human and mouse tyrosinases is 2.68 Å, with a TM-score of 0.855 and sequence identity of 86.7%, indicating similarity between the two mammalian tyrosinases (Figure S2). The limited structural similarity in mushroom and mammalian tyrosinases necessitates the experimental validation of whether hydroxamate-containing inhibitors inhibit mammalian tyrosinases [51].

The melanin quantification assay and the cell-lysate-based activity assay were used for confirming the inhibition of mammalian tyrosinases. Unfortunately, quantifying melanin content using hydroxamate-containing molecules is not feasible because anticancer agents affect cell viability. Therefore, we evaluated tyrosinase activities using B16F10 cell lysates in a small-molecule-concentration-dependent manner.

**Figure 5:**
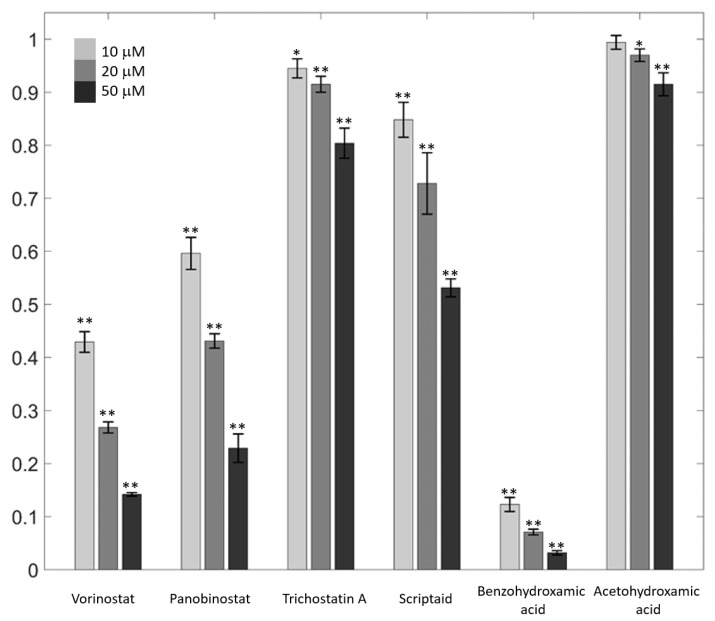
Mammalian-tyrosinase inhibition. B16F10 cell lysates were used to evaluate inhibition against different concentrations of hydroxamate-containing tyrosinase inhibitors. Data are expressed as values relative to those of untreated control cells. Light gray, gray, and dark gray indicate data for inhibitor concentrations of 10, 20, and 50 μM, respectively. Error bars indicate standard deviations for three repeated independent experiments. * and ** indicate statistically significant differences at the levels of *p* < 0.05 and *p* < 0.01, respectively, compared with the controls using *t*-tests.

All molecules that inhibited mushroom tyrosinase decreased tyrosinase activities concentration-dependently at 10, 20, and 50 μM (Figure 5). Notably, benzohydroxamic acid exhibited the most potent inhibition, decreasing the activity by approximately 97% at 50 μM (Figure 5). This finding is entirely consistent with the results of an enzyme-based assay observed with mushroom tyrosinase, suggesting that the molecules are inhibitors for mammalian tyrosinase.

### 3.5. Cheminformatics and Docking Studies Suggested the Binding Modes of Hydroxamate-Containing Inhibitors

The binding mode of hydroxamate-containing inhibitors in complex with tyrosinase is insightful information. Unlike HDACs, which possess a zinc atom, the catalytic center of tyrosinase consists of two copper atoms. To evaluate how hydroxamic acid recognized the dimetal, we searched for hydroxamate-containing molecules among protein–ligand-complex structures using the PDBbind database [29]. There were 99 hydroxamate-containing molecules <500 Da in mass. Of these, nine were found to bind to metalloenzymes with a dimetal catalytic center. The PDB IDs (metal and code of hydroxamate-containing ligand) of them are 1EBG (Mg^2+^ and PAH), 1IGB (Zn^2+^ and IPO), 2GYI (Mg^2+^ and HYA), 2PU1 (Zn^2+^ and FSG), 4R7M (Zn^2+^ and 3MW), 4ZX8 (Zn^2+^ and 4TY), 4ZY1 (Zn^2+^ and 4U5), 5D29 (Zn^2+^ and 5Q1), and 5ELY (Zn^2+^ and 5PU) (Figure S3). Interestingly, all the proteins are hydrolases; 1IGB, 4R7M, 4ZX8, and 4ZY1 are aminopeptidases; 1EBG and 2PU1 are enolases; 2GYI is a xylose isomer; and 5D29 and 5ELY are carboxypeptidases.

We performed docking simulation studies to predict the bound poses of hydroxamate-containing inhibitors with tyrosinase. We first checked which docking software could faithfully reproduce the crystal poses. Three pieces of software, DOCK3.7 [31,32], Fred [33,34], and Glide-SP [35,36], were used because they can handle intermolecular interactions between small organic molecules and metals. Fred and Glide-SP reproduced all nine poses of the crystal ligands within 2 Å. The manual inspection of the poses suggested more faithful geometries in the results of Glide-SP; therefore, we used Glide-SP for the studies of the docking between hydroxamate-containing inhibitors and mushroom tyrosinase (PDB ID: 2Y9X).

**Figure 6:**
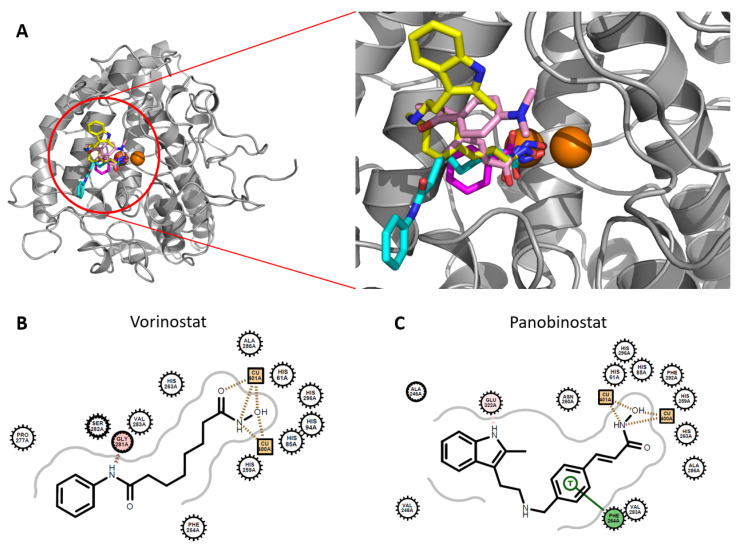
Predicted poses of hydroxamate-containing HDAC inhibitors. (**A**) Overlaid poses of hydroxamate-containing molecules docked to mushroom tyrosinase. Molecules in cyan, yellow, pink, and magenta indicate vorinostat, panobinostat, trichostatin A, and benzohydroxamic acid, respectively. Brown spheres represent copper atoms. Dockings were performed using Glide-SP with mushroom tyrosinase (PDB ID: 2Y9X) as a template. (**B**,**C**) 2D diagrams of docked poses for vorinostat and panobinostat. The OpenEye Grapheme package was used to analyze and visualize intermolecular interactions. For the definition, see Figure S3.

Figure 6 shows overlaid docked poses of hydroxamate-containing inhibitors, vorinostat, panobinostat, trichostatin A, and benzohydroxamic acid, against mushroom tyrosinase. The hydroxamate moieties were found to face toward the space between the two copper atoms. Although benzohydroxamic acid somewhat deviates, the other hydroxamates are well overlaid, supporting the reliability of our approach. The patterns are similar to those found in hydroxamates and dimetal–enzyme-complex structures. Notably, most of the intermolecular contacts occur between the copper atoms and hydroxamic acid parts. This explains the necessity of the hydroxamate moiety for inhibiting tyrosinase.

Nevertheless, we cannot exclude the possibility that the docked poses are inaccurate. Unlike those in di-Zn and di-Mg cases, the surroundings and oxidation states of di-Cu in tyrosinase vary depending on the catalytic cycle. For instance, the interatom distance between two coppers switches from 2.9 to 4.9 Å in the met, deoxy, and oxy states [52,53,54]; however, there is no experimental clue as to which state hydroxamate-containing inhibitors bind, making docking simulation challenging. A detailed understanding of inhibition at the atomic level may necessitate experimental structure determination of complexes between tyrosinase and new inhibitors.

## 4. Discussion

Hydroxamic acid is a bioisostere of carboxylic acid [55], a representative MBG. The chelator, ethylenediaminetetraacetic acid, shows the role of carboxylic acid in forming a coordinate bond with the metal. We have reported that tetrazole and triazole, two bioisosteres of carboxylic acid, are novel MBGs that interact with the dicopper of tyrosinase [13]. Other bioisosteres of carboxylic acid may also act as MBGs for inhibiting tyrosinase; however, the data on experimentally confirmed MBGs for tyrosinase are limited.

Accumulating pharmaceutical and toxicological data have uncovered the polypharmacological networks of bioactive compounds. Nevertheless, practically effective strategies for finding new modulators or other targets have been less reported. We have reported novel tyrosinase and laccase inhibitors by examining cross-inhibition between copper-containing enzymes [21]. Adopting the method of comprehensively cross-examining inhibitors of tyrosinase and other metalloenzymes, including HDACs, may lead to the discovery of tyrosinase inhibitors with new MBGs.

The reported inhibitory activities of vorinostat for human HDACs range from 1 to 25 nM in terms of K_i_ or IC_50_ values; panobinostat inhibits human HDACs with values of 0.6 to 25 nM [41,56]. Therefore, tyrosinase is off-target for vorinostat and panobinostat. By contrast, the quantified inhibition by benzohydroxamic acid of HDACs ranges from 110 nM to 8 μM in terms of K_i_ or IC_50_ values [41,56]. Benzohydroxamic acid can inhibit other metalloenzymes, such as carbonic anhydrase [57], indoleamine 2,3-dioxygenase [58], arachidonate 5-lipoxygenase [59], and mandelate racemase [60], in the μM range. This indicates that benzohydroxamic acid is more potent in inhibiting tyrosinase despite its lower selectivity. Of the ChEMBL-registered tyrosinase inhibitors, the reportedly most potent ones are CHEMBL1408767 [61] and CHEMBL4463984 [62], with respective IC_50_ values of 10 and 11 nM. Both are substrate-mimicking polyphenol compounds. We also reported that a tetrazole compound, **8b**, had a K_i_ of 11 nM [13] (Figure S4). The inhibitory activity of benzohydroxamic acid (7 nM K_i_) is comparable to that of these compounds within experimental uncertainty, highlighting benzohydroxamic acid as one of the most potent tyrosinase inhibitors.

In this study, our experiments mainly focused on quantitatively characterizing the inhibition against mushroom tyrosinase. However, studies have described selective inhibitors for human and mushroom tyrosinases [51,63,64]. Significant differences in the 3D structures of tyrosinases from evolutionarily remote species may enable the development of species-selective inhibitors. The quantification of species-dependent activities and selective potentiation of the current hydroxamate-containing inhibitors for mammalian tyrosinases will be a topic deserving further study.

Several studies have reported a correlation between tyrosinase and cancer [65,66]. Mainly, melanoma cells show increased levels of tyrosinase expression. However, whether the increased activity of tyrosinase is the direct cause of the transformation into melanoma is unclear. Nevertheless, the activity of tyrosinase, a key enzyme for melanin biosynthesis, will influence cancer signaling because melanin production can contribute to diverse signaling pathways. Considering that the use of hydroxamate-containing HDAC inhibitors is one of the available options for treating melanomas [67,68,69], one must be cautious about the potential effects of tyrosinase inhibition. Our data will be a meaningful addition in this direction.

## Figures and Tables

**Table 1:** Molecules and their inhibition for mushroom tyrosinase.

Compound	ZINC ID	MW	cLogP	K_i_
Vorinostat	ZINC1543873	264	2.47	257 ± 26 nM
Panobinostat	ZINC22010649	349	3.33	40 ± 5 nM
Trichostatin A	ZINC100014731	302	2.58	1.0 ± 0.1 μM
Scriptaid	ZINC3873638	326	2.50	180 ± 21 nM
Benzohydroxamic acid	ZINC4701351	137	1.38	7 ± 1 nM
Acetohydroxamic acid	ZINC4658603	75	−0.49	38 ± 8 μM
N-Hydroxyacetamide	ZINC4692578	75	−1.54	>1 mM
Romidepsin	ZINC100371951	542	1.99	>500 μM
Valproic acid	ZINC3008621	144	2.29	>500 μM
Suberohydroxamic acid	ZINC3873635	204	0.34	1.0 ± 0.2 μM
Butyrylhydroxamic acid	ZINC4809144	103	0.29	32 ± 5 μM
PTU *	ZINC3875720	152	1.34	62 ± 3 nM
Tropolone *	ZINC392003	122	0.75	43 ± 3 nM

ZIND ID is the ID in ZINC database [41]. MW and cLogP mean molecular weight and calculated LogP. K_i_ is the inhibitory constant converted from IC_50_. The value following ± is standard deviation calculated using Monte-Carlo simulation of 500 cycles with at least 5% uncertainty in experimental data. * indicates known tyrosinase inhibitors used as positive controls.

## Data Availability

Data is contained within the article.

## Supplementary Materials

The following are available online at www.mdpi.com/article/10.3390/antiox11020280/s1. Figure S1: Known hydroxamate-containing tyrosinase inhibitors, Figure S2: Comparison of mushroom, and AlphaFold-derived human and mouse tyrosinases, Figure S3: 2D diagrams for hydroxamate-containing inhibitors and di-metal metalloenzymes, Figure S4. Known potent tyrosinase inhibitors.
