# Supplementary material for: Hydroxamic Acid as a Potent Metal-Binding Group for Inhibiting Tyrosinase
Source: Antioxidants (Basel). 2022 Jan 29;11(2):280. doi: 10.3390/antiox11020280 (PMC8868331; doi:10.3390/antiox11020280)
Supplement: Supplementary file 1 [file antioxidants-11-00280-s001.zip › antioxidants-1578234-supplementary.pdf]

Supplementary materials

Hydroxamic Acid as a Potent Metal-Binding Group for Inhibiting Tyrosinase

Joonhyeok Choi[†], Trilok Neupane[†], Rishiram Baral and Jun-Goo Jee*

Research Institute of Pharmaceutical Sciences, College of Pharmacy, Kyungpook National University, 80 Daehak-ro, Buk-gu, Daegu 41566, Republic of Korea

* Correspondence: jjee@knu.ac.kr; Tel.: +82-53-950-8568
[†] These authors contributed equally to this work.

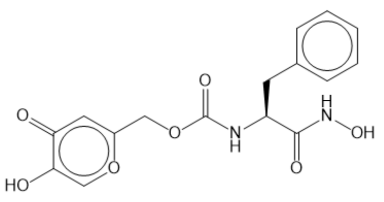

CHEMBL2332235

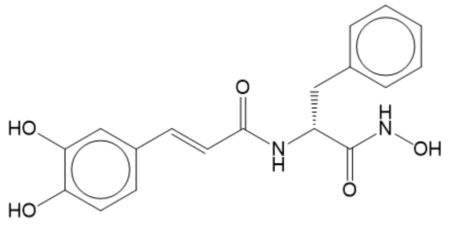

CHEMBL1825209

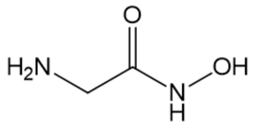

Gly-NHOH

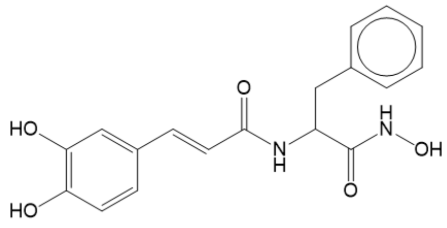

CA-Phe-NHOH

Figure S1. Known hydroxamate-containing tyrosinase inhibitors. ChEMBL database [1,2] includes CHEMBL2332235 and CHEMBL1825209. Gly-NHOH [3] and CA-Phe-NHOH [4,5] are inhibitors found by extensive publication survey.

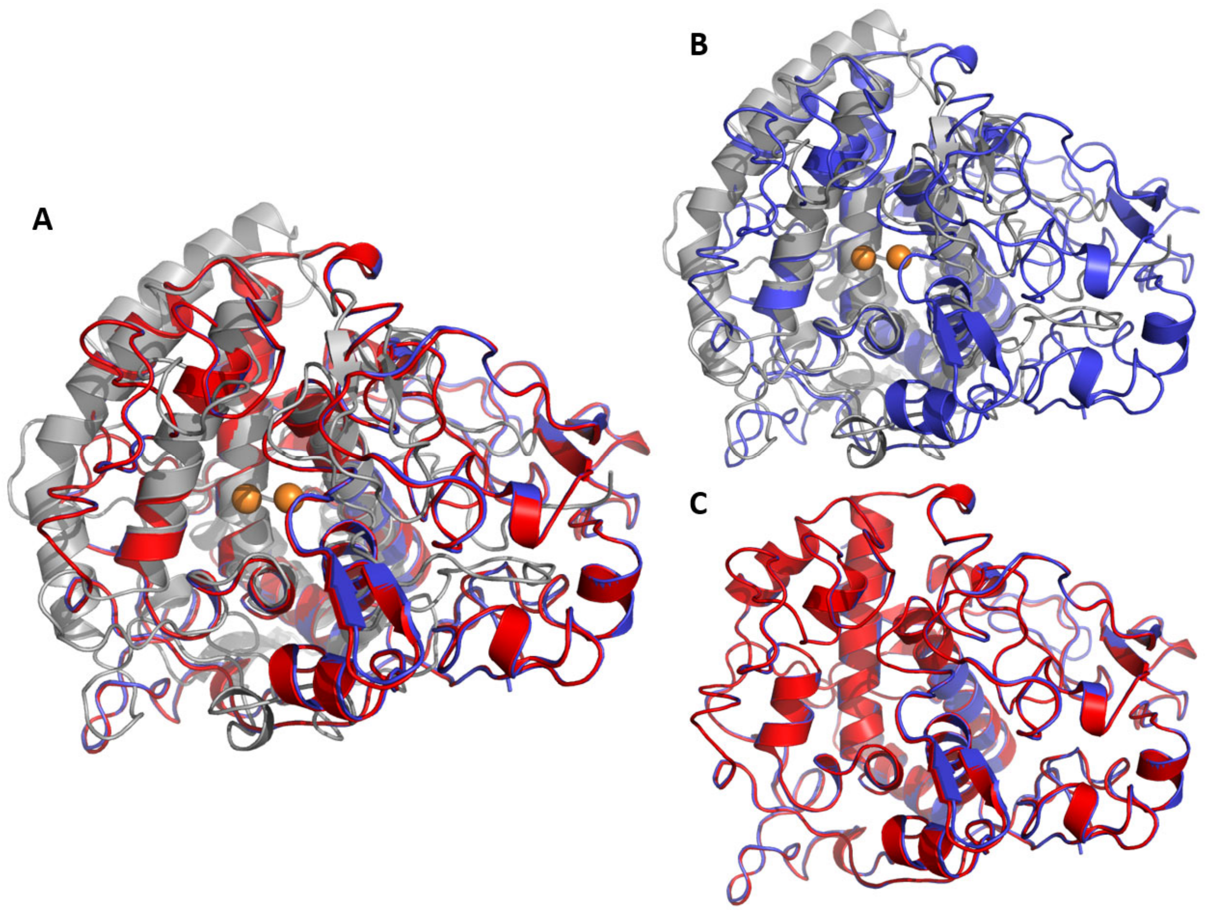

Figure S2. Comparison of mushroom, and AlphaFold-derived human and mouse tyrosinases. Mushroom tyrosinase (PDB ID: 2Y9X), and human (UniProt ID: P14679) and mouse (P11344) tyrosinase are extracted from PDB and AlphaFold databases. Gray, blue, and red colors indicate mushroom, human, and mouse tyrosinases, respectively. N- and C-terminal parts of human and mouse tyrosinases are omitted for clarity. (**A**) Overlay of three tyrosinase structures**.** (**B**) Overlay of mushroom and human tyrosinase structures. (**C**) Overlay of human and mouse tyrosinase structures.

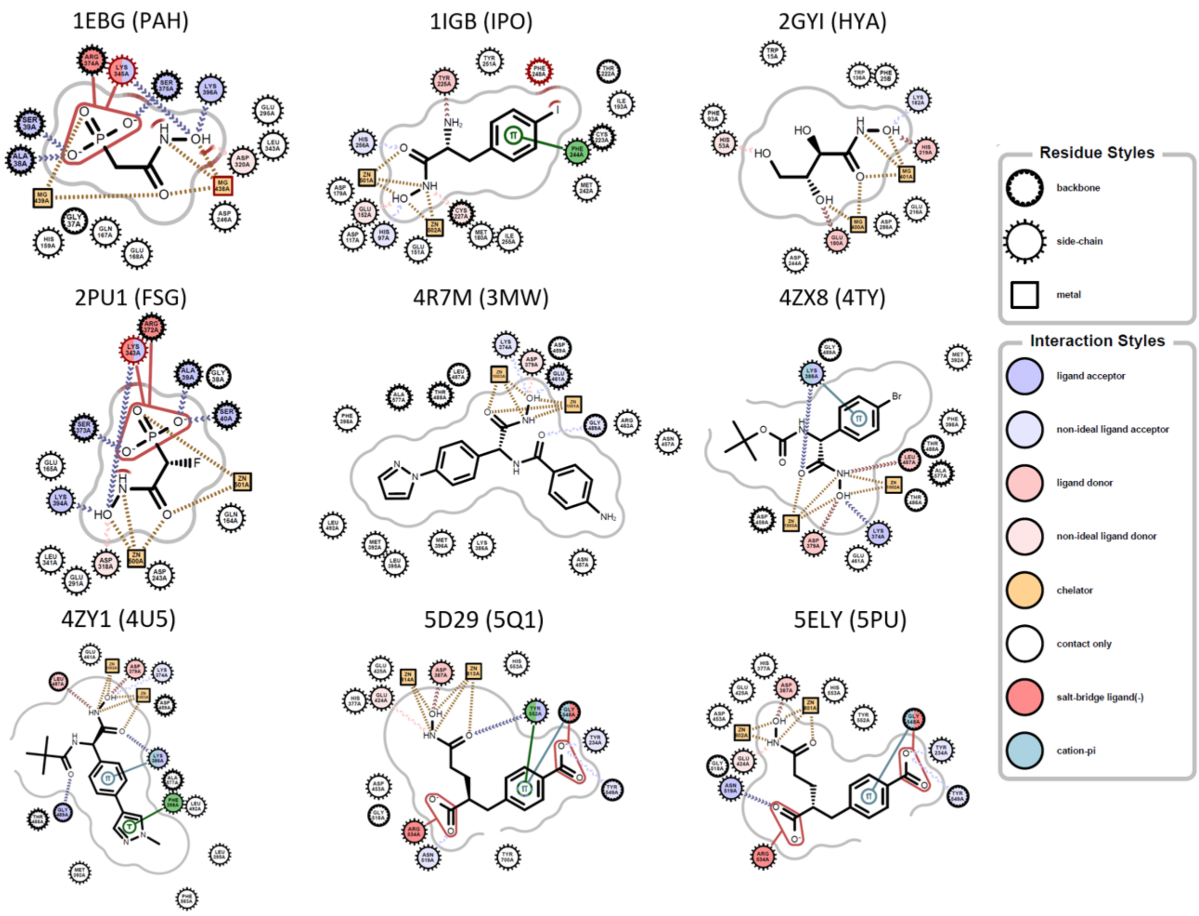

Figure S3. 2D diagrams for hydroxamate-containing inhibitors and di-metal metalloenzymes. PDBbind database [6] is employed to find nine hydroxamate-containing molecules complexed with the metalloenzymes having a di-metal catalytic center. The PDB IDs (code of hydroxamate-containing ligand) of them are 1EBG (PAH), 1IGB (IPO), 2GYI (HYA), 2PU1 (FSG), 4R7M (3MW), 4ZX8 (4TY), 4ZY1 (4U5), 5D29 (5Q1), and 5ELY (5PU). The OpenEye Grapheme package is employed to analyze and visualize the intermolecular interactions. Definition of the interactions is drawn on the right side.

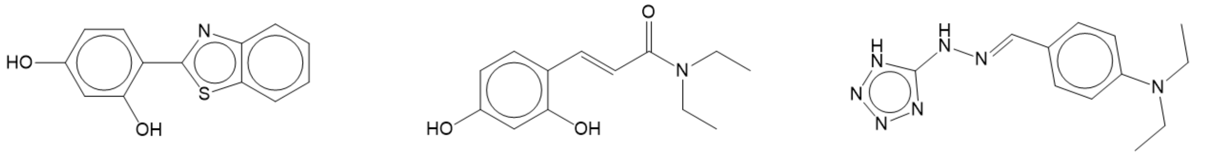

CHEMBL1408767 CHEMBL4463984 **8b**

Figure S4. Known potent tyrosinase inhibitors. The reported IC_{50} values for CHEMBL1408767 [7] and CHEMBL4463984 [8] are 10 and 11 nM, respectively. The K_i value for **8b** is 11 nM [9].

References

1. Mendez, D.; Gaulton, A.; Bento, A.P.; Chambers, J.; De Veij, M.; Felix, E.; Magarinos, M.P.; Mosquera, J.F.; Mutowo, P.; Nowotka, M., et al. ChEMBL: towards direct deposition of bioassay data. *Nucleic acids research* **2019**, *47*, D930-D940, doi:10.1093/nar/gky1075.
2. Gaulton, A.; Hersey, A.; Nowotka, M.; Bento, A.P.; Chambers, J.; Mendez, D.; Mutowo, P.; Atkinson, F.; Bellis, L.J.; Cibrian-Uhalte, E., et al. The ChEMBL database in 2017. *Nucleic acids research* **2017**, *45*, D945-D954, doi:10.1093/nar/gkw1074.
3. Lin, Y.S.; Wu, W.C.; Lin, S.Y.; Hou, W.C. Glycine hydroxamate inhibits tyrosinase activity and melanin contents through downregulating cAMP/PKA signaling pathways. *Amino acids* **2015**, *47*, 617-625, doi:10.1007/s00726-014-1895-8.
4. Kwak, S.Y.; Yang, J.K.; Choi, H.R.; Park, K.C.; Kim, Y.B.; Lee, Y.S. Synthesis and dual biological effects of hydroxycinnamoyl phenylalanyl/prolyl hydroxamic acid derivatives as tyrosinase inhibitor and antioxidant. *Bioorganic & medicinal chemistry letters* **2013**, *23*, 1136-1142, doi:10.1016/j.bmcl.2012.10.107.
5. Kwak, S.Y.; Lee, S.; Choi, H.R.; Park, K.C.; Lee, Y.S. Dual effects of caffeoyl-amino acidyl-hydroxamic acid as an antioxidant and depigmenting agent. *Bioorganic & medicinal chemistry letters* **2011**, *21*, 5155-5158, doi:10.1016/j.bmcl.2011.07.064.
6. Liu, Z.; Li, Y.; Han, L.; Li, J.; Liu, J.; Zhao, Z.; Nie, W.; Liu, Y.; Wang, R. PDB-wide collection of binding data: current status of the PDBbind database. *Bioinformatics* **2015**, *31*, 405-412, doi:10.1093/bioinformatics/btu626.
7. Ha, Y.M.; Park, J.Y.; Park, Y.J.; Park, D.; Choi, Y.J.; Kim, J.M.; Lee, E.K.; Han, Y.K.; Kim, J.A.; Lee, J.Y., et al. Synthesis and biological activity of hydroxy substituted phenyl-benzo[d]thiazole analogues for antityrosinase activity in B16 cells. *Bioorganic & medicinal chemistry letters* **2011**, *21*, 2445-2449, doi:10.1016/j.bmcl.2011.02.064.
8. Ullah, S.; Kang, D.; Lee, S.; Ikram, M.; Park, C.; Park, Y.; Yoon, S.; Chun, P.; Moon, H.R. Synthesis of cinnamic amide derivatives and their anti-melanogenic effect in alpha-MSH-stimulated B16F10 melanoma cells. *European journal of medicinal chemistry* **2019**, *161*, 78-92, doi:10.1016/j.ejmech.2018.10.025.
9. Choi, J.; Choi, K.E.; Park, S.J.; Kim, S.Y.; Jee, J.G. Ensemble-Based Virtual Screening Led to the Discovery of New Classes of Potent Tyrosinase Inhibitors. *Journal of chemical information and modeling* **2016**, *56*, 354-367, doi:10.1021/acs.jcim.5b00484.
